# Supplementary material for: Complexome profiling on the Chlamydomonas lpa2 mutant reveals insights into PSII biogenesis and new PSII associated proteins
Source: J Exp Bot. 2021 Aug 26;73(1):245–62. doi: 10.1093/jxb/erab390 (PMC8730698; doi:10.1093/jxb/erab390)
Supplement: erab390_suppl_Supplementary_Dataset_S1 [file erab390_suppl_supplementary_dataset_s1.zip › Supplemental Dataset 1 - Excel List and all profiles/plots/SCLA1_Cre03.g193850.html]

Trivial name: SCLA1  
  
Euclidean distance: 37478.70  
Mean Intensity (WT): 10935.30  
Mean Intensity (Mut): 9859.41  
Distance: 3.43  
  
MapMan: TCA / organic transformation.TCA.succinyl-CoA ligase  
  
p value of intensity sums Welch test: 0.8357
